# Case Report: An NTRK1 fusion-positive embryonal rhabdomyosarcoma: clinical presentations, pathological characteristics and genotypic analyses

**DOI:** 10.3389/fonc.2023.1178945

**Published:** 2023-04-28

**Authors:** Na-Mei Li, Shi-He Jiang, Peng Zhou, Xiao-Hong Li

**Affiliations:** Department of Pathology, The Second Xiangya Hospital, Central South University, Changsha, China

**Keywords:** embryonal rhabdomyosarcoma, NTRK1 fusion, case report, pathological features, next generation sequencing (NGS)

## Abstract

Rhabdomyosarcoma (RMS) is a prevalent form of soft tissue sarcoma that primarily affects children. Pediatric RMS is characterized by two distinct histological variants: embryonal (ERMS) and alveolar (ARMS). ERMS is a malignant tumor with primitive characteristics resembling the phenotypic and biological features of embryonic skeletal muscles. With the widespread and growing application of advanced molecular biological technologies, such as next-generation sequencing (NGS), it has been possible to determine the oncogenic activation alterations of many tumors. Specifically for soft tissue sarcomas, the determination of tyrosine kinase gene and protein related changes can be used as diagnostic aids and may be used as predictive markers for targeted tyrosine kinase inhibition therapy. Our study reports a rare and exceptional case of an 11-year-old patient diagnosed with ERMS, who tested positive for MEF2D-NTRK1 fusion. The case report presents a comprehensive overview of the clinical, radiographic, histopathological, immunohistochemical, and genetic characteristics of a palpebral ERMS. Furthermore, this study sheds light on an uncommon occurrence of NTRK1 fusion-positive ERMS, which may provide theoretical basis for therapy and prognosis.

## Introduction

Rhabdomyosarcoma (RMS) is a common malignant tumor, accounting for 50% of all soft tissue sarcomas in children. It originates from the embryonic mesenchyme precursor of striated muscle and fails to undergo terminal differentiation (1). Based on the histopathologic and molecular features, RMS is categorized into four primary subtypes, which are embryonal (ERMS), alveolar (ARMS), pleomorphic (PRMS), and spindle cell/sclerosing (SpRMS) (2). Currently, the diagnosis of RMS mainly relies on morphology (rhabdomyoblastic differentiation) and immunohistochemistry (IHC) (expression pattern of Myogenin and MYO-D1) (3). ERMS represents the most prevalent subtype of RMS and is imparted with a favorable prognosis; whereas, ARMS is known to exhibit a more aggressive clinical course and is often associated with a higher incidence of metastasis (2). The cornerstone of treatment comprises of multi-agent chemotherapy along with local intervention strategies, including surgery and/or radiotherapy, as required (3, 4).

With widespread applications of NGS, genetic testing in RMS diagnosis have delineated the mechanism of oncogenesis. The majority of ARMS harbor PAX-FOXO gene fusions owing to chromosomal translocations, mostly involving in PAX3-FOXO1 and PAX7-FOXO1 (5), and a small subset expressing PAX3-FOXO4 or PAX3-NOXA1 (6). As a transcription factor, the chimeric protein PAX-FOXO drives the expression of oncogenic genes. Molecular ancillary testing in ARMS even proposes a challenge for morphological classification: fusion-positive ARMS showing worse survival than fusion-negative subtype, irrespective of histopathologic features. Moreover, fusion-negative ARMS exhibits the molecular profile and clinical outcome that are analogous to the ERMS subtype (7). Application of ARMS fusion status in the risk stratification is popular in clinical trials (8, 9).

SpRMS was initially established as a distinct entity in the WHO 2013 classification of soft tissue and bone neoplasms (10). Morphologically, bland spindle cell and extensive hyalinized matrix are the outstanding features of spindle cell RMS and sclerosing RMS separately. The discovery of several significant genes associated with SpRMS has greatly deepened our comprehension of the biological processes underlying SpRMS, as well as indicated that it is a heterogeneous group of tumors, molecularly classifying it into four categories (1): infantile/congenital SpRMS harboring NCOA2 or VGLL2 gene fusions (11); (2) SpRMS occurring in the adult and pediatric which show MYOD1 gene mutations (12); (3) SpRMS with EWSR1/FUS–TFCP2 gene fusion, predilection for intraosseous locations (13, 14); (4) SpRMS with no known recurrent abnormalities. MYOD1 mutated SpRMS and SpRMS with EWSR1/FUS–TFCP2 gene fusion both behave aggressively and have a poor prognosis (15, 16). Molecular classification of SpRMS facilitates prognostic stratification.

Genetic analyses typically have indicated that ERMS is a biologically heterogeneous group of disorders, involving in aneuploidy and gene mutation including RAS genes (HRAS, KRAS, and NRAS) (17, 18), FGFR4 (19, 20), PIK3CA, NF1 and FBXW7 (21, 22). It has been observed in 1996 that ERMS exhibits the gain of multiple chromosomes, with notable instances on chromosomes 2, 7, 8, 12, 13, 17, 18, and 19, while concurrently demonstrating the loss of chromosomes 10, 14, 15, and 16 (23). In both fusion gene-negative ARMS and ERMS, frequent alterations can be observed in whole chromosome copy numbers, particularly, the amplification in chromosome 8 (24). At present, few literatures have reported that ERMS tumors harbor gene rearrangement. Here, we find a rare case of ERMS, harboring neurotrophic receptor tyrosine kinase 1 (NTRK1) gene rearrangement, which can broaden our understanding of ERMS genotype and maybe provide treatment options.

## Case presentation

**Figure 1:**
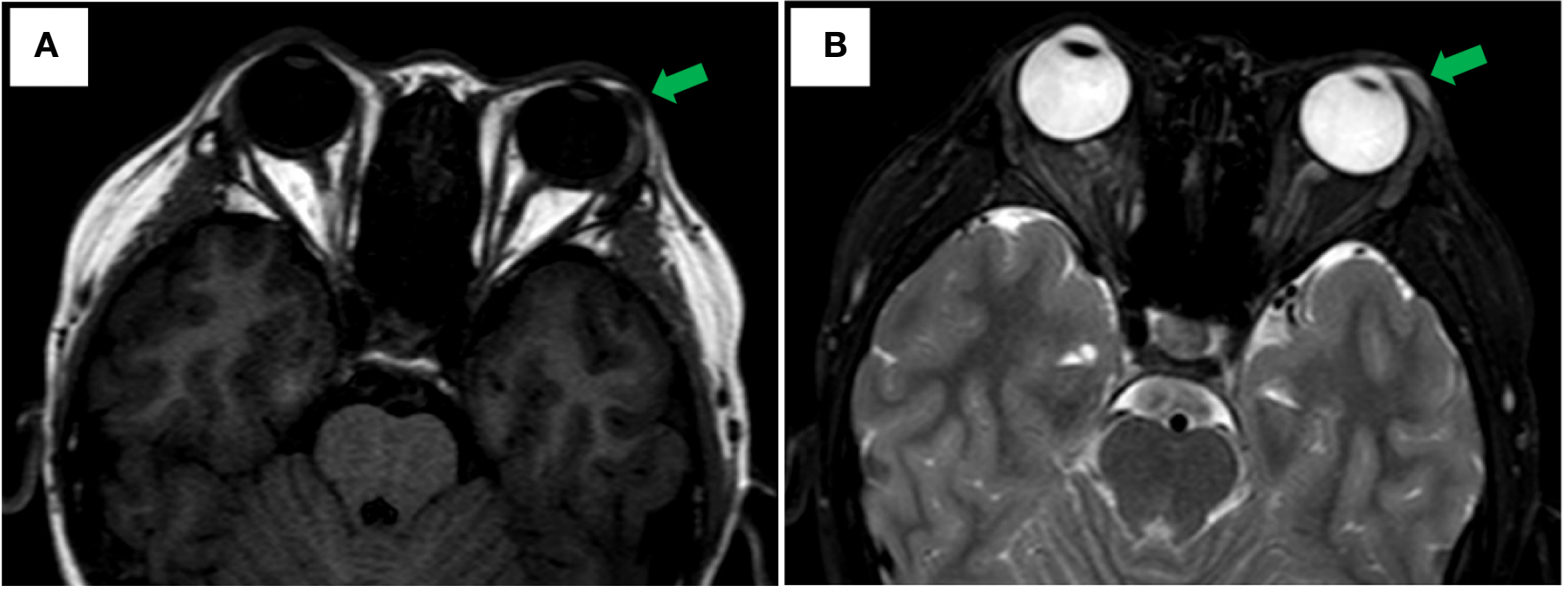
Axial MRI Imaging. **(A)** Well-circumscribed fusiformmass is noted in the left lower eyelid appearing hypointense on T1 weighted image with green arrowhead. **(B)** T2-weighted showing hyperintense with green arrowhead.

**Figure 2:**
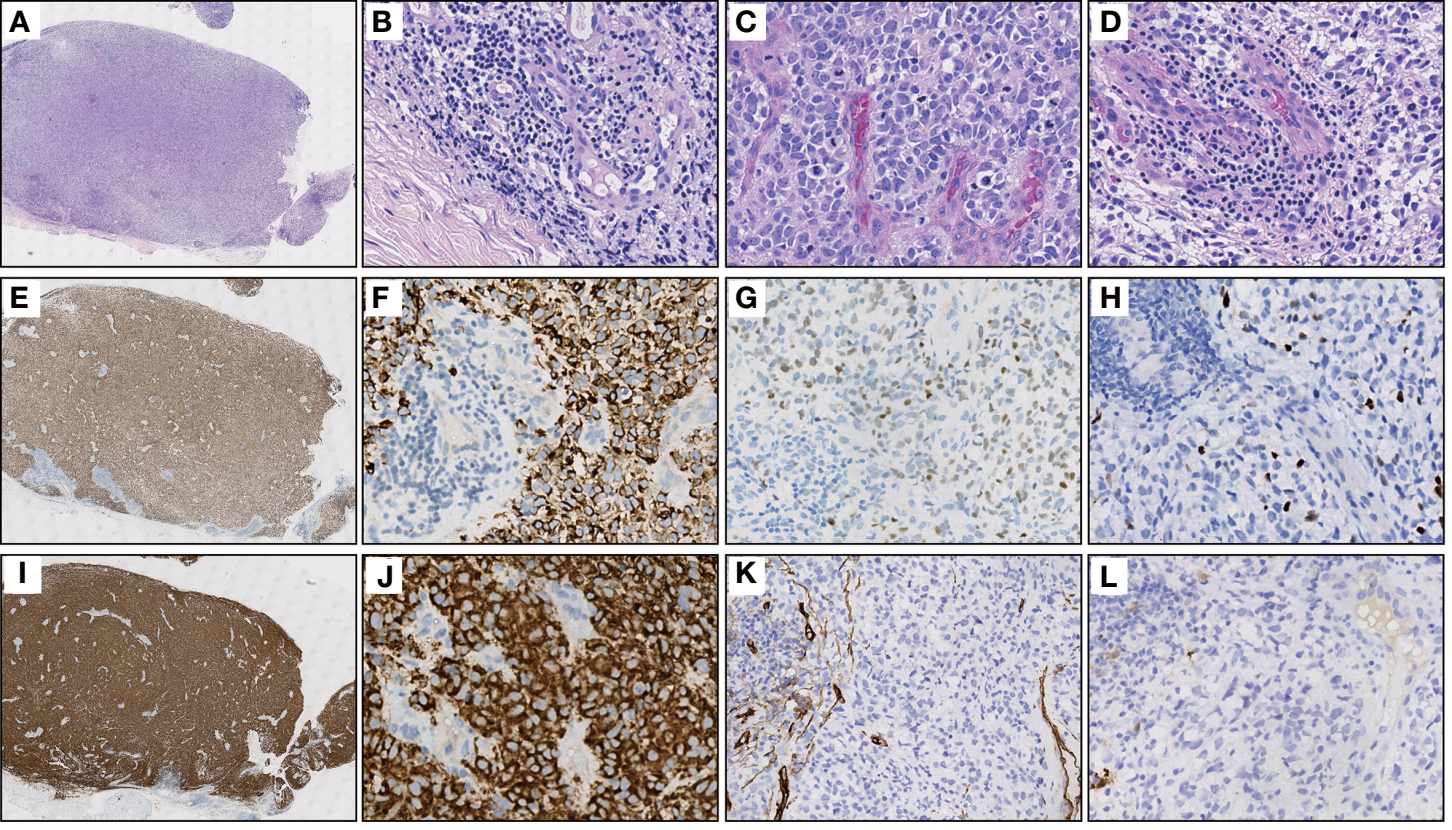
Representative H&E and IHC stainings of serial sections. **(A)** Extra-low power H&E image shows a well-demarcated mass entirely, surrounded by fibrous pseudocapsule. **(B)** High power view of A gets more detail about the pseudocapsule, simultaneously, demonstrates hyperendothelial vessels and lymphocytes that aggregated around these vessels. **(C)** The tumor is primarily composed of primitive round cells with scant cytoplasm and numerous mitosis. **(D)** Focally shows clear cytoplasm and lymphocytes aggregating around the hyperendothelial vessels. **(E)** Diffuse Desmin staining in the same power view of **(A, F)** A high power view of E showing cytoplasmic positive of Desmin, while the nonmuscular hyperendothelial vessels are negative for Desmin. **(G, H)** Focal positive immunohistochemical staining of tumor cells for MYOD1 and Myogenin respectively. **(I, J)** The immunohistochemical staining for TrkA/B/C expression was performed using pan-Trk (clone EPR17341, Roche/Ventana). There is a strong diffuse immunoreactivity for pan-Trk in cytoplasm of tumor cells. **(K, L)** Negative IHC reaction for CD34 and S100, respectively.

An 11-year-old male youngster reported a 20-day history of eyelid mass, which is painless and non-pruritic, as well as no skin ulceration. MRI scan revealed a subcutaneous mass (1.2 × 0.5 cm) in the left lower eyelid, which was well demarcated indicating a pre-operative clinical impression of skin benign tumor (**Figures 1A, B**). After surgical excision of subcutaneous tumor, samples were micro-evaluated in our department, and presented as monomorphous population of primitive cells with abundant mitosis and minimal cytoplasm (**Figures 2A, C**). The mass was surrounded by a continuous fibrous pseudocapsule (**Figures 2A, B**). Hyperendothelial vessels lied in maliglant tumor cells, with small lymphocytes surrounding these vessels (**Figures 2B, D, F, G, H, K, L**). Based on extensive H&E staining observation, there is no evidence of rhabdomyoblastic differentiation.

**Figure 3:**
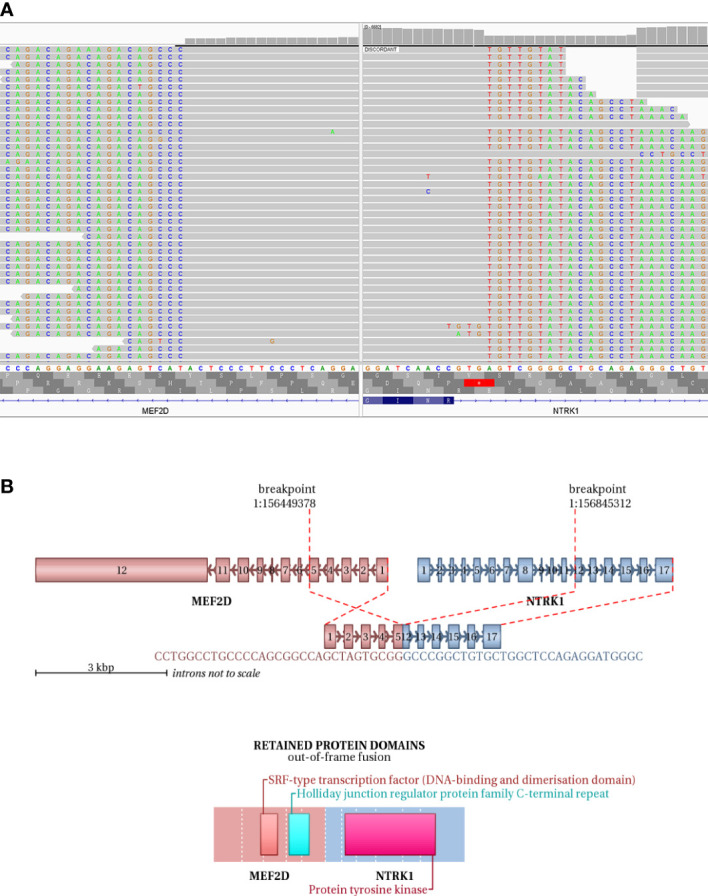
NGS testing of the tumor to validate NTRK1 fusion. **(A)** DNA-based NGS testing of the pan-tumor related 1021-Genes Panel is used to demonstrate the MEF2D-NTRK1 (EX5:EX12) fusion in formalin-fixed paraffin-embedded tissue sample. **(B)** Schematic representation of the predicted chimeric protein in RNA-based NGS assay with the 555-Genes Panel in formalin-fixed paraffin-embedded tissue sample.

IHC demonstrated striated muscle differentiation (diffusely positive for DES, MYOD1 and Myogenin showing patchy staining) (**Figures 2E–H**), which led to reliable diagnosis of ERMS in the case. DNA-based NGS revealed MEF2D-NTRK1 (EX5:EX12) fusion (**Figure 3A**), and NTRK1 amplification, along with multiple genes amplifications (CDK6, PMS2, MET, EGFR, BRAF, MLL3, MYC, FGFR1, WRN, EXT1, NBN, RECQL4) and deletions (FLT4, MAP2K2, DOT1L, STK11, GNA11) (**Table 1**). These copy number variations involve chromosomes 7, 8 and 19. Next, RNA-based NGS also revealed MEF2D-NTRK1 (EX5:EX12) fusion (**Figure 3B**). IHC detection of Pan-Trk is a dependable and effective method for identifying NTRK fusions (25). In consideration of MEF2D-NTRK1 fusion detected by NGS, follow-up confirmatory IHC staining of pan-Trk was performed, as expected, it demonstrated diffusely strong positive in cytoplasm (**Figures 2I, J**). In terms of differential diagnosis in IHC, CD34 and S-100 were negative (**Figures 2K, L**). To sum up, the case is a rare ERMS with NTRK1 fusion positive, not an NTRK-rearranged spindle cell tumor.

**Table 1:** Genetic mutations of DNA-based next generation sequencing (NGS) in the case.

Mutation Genes	Location	Transcript Version	Variant types	Copy number/ Mutation frequency
NTRK1	1q23.1	NM_002529.3	all exon, amplification	13.0
CDK6	7q21.2	NM_001145306.1	all exon, amplification	4.8
PMS2	7p22.1	NM_000535.5	all exon, amplification	4.0
MET	7p31.2	NM_000245.2	all exon, amplification	3.6
EGFR	7p11.2	NM_005228.3	all exon, amplification	3.6
BRAF	7q34	NM_004333.4	all exon, amplification	3.6
MLL3	7q36.1	NM_170606.2	all exon, amplification	3.6
MYC	8q24.21	NM_002467.4	all exon, amplification	8.2
FGFR1	8p11.23	NM_023110.2	all exon, amplification	7.0
WRN	8p12	NM_000553.4	all exon, amplification	6.8
EXT1	8q24.11	NM_000127.2	all exon, amplification	6.6
NBN	8q21.3	NM_002485.4	all exon, amplification	6.0
RECQL4	8q24.3	NM_004260.3	all exon, amplification	5.4
FLT4	5q35.3	NM_182925.4	all exon, deletion	1.2
MAP2K2	19p13.3	NM_030662.3	all exon, deletion	1.2
DOT1L	19p13.3	NM_032482.2	all exon, deletion	1.0
STK11	19p13.3	NM_000455.4	all exon, deletion	1.0
GNA11	19p13.3	NM_002067.2	all exon, deletion	1.0
MEF2D-NTRK1	1q22; 1q23.1	NM_005920.2; NM_002529.3	Fusion (EX5:EX12)	49.7%

## Discussion

RMS is a frequently occurring soft tissue sarcoma in children, constituting over 50% of all cases (3). ERMS, the most common subtype, is an unsophisticated and malignant neoplasm of soft tissue that exhibits characteristics similar to those of embryonic skeletal muscle, both phenotypically and biologically (26). Histologically, ERMS features a proliferation of undifferentiated mesenchymal cells that exhibit round or spindle-shaped morphologies, mixed with a varying number of rhabdomyoblasts and interspersed with zones of stroma that are loose, myxoid, and paucicellular (27). ERMS is typified by the presence of oval to spindle-shaped primitive cells with minimal cytoplasm. These cells can be arranged in compact sheets or surrounded by a loose, myxoid background. Certain regions of ERMS display a small, blue, and round morphology. As these cells undergo differentiation, they gradually exhibit an increased eosinophilic cytoplasm and adopt elongated shapes that are described variously as “tadpole”, “strap”, and “spider” cells, indicating the presence of rhabdomyoblastic differentiation (28). Desmin is the leading diagnostic marker, commonly demonstrating diffuse staining. In general, ERMS features a patchy positivity for Myogenin and MYO-D1, and there are apparent divergences in Myogenin staining patterns between ERMS and ARMS. Specifically, ARMS usually displays a diffuse Myogenin staining, while in ERMS, the staining frequently appears patchy. Differences in Myogenin staining patterns have also been observed between ERMS and ARMS. ARMS tends to show diffuse staining for Myogenin, whereas the staining is often patchy in ERMS. In our report, IHC expression patterns of Desmin, Myogenin, and MYO-D1 were consistent with the immunological phenotype of ERMS.

Genotypic analyses of ERMS and fusion-negative ARMS typically reveal aneuploidy characterized by numerous copy number gains and losses (7). The ERMS tumors have been demonstrated the gain of whole or most of distinct chromosomes, particularly chromosomes 2, 13, 12, 8, 7, 17, 18, and 19, along with the loss of chromosomes 16, 10, 15, and 14 (23). ERMS is linked to distinct genetic changes, which encompass chromosomal gains and losses resulting in aneuploidy. Additionally, ERMS involves modifications in RAS family genes (HRAS, NRAS, KRAS), FGFR4, PIK3CA, NF1, and FBXW7 (28–30). In ARMS, identifying PAX3-FOXO1 and PAX7-FOXO1 gene fusions is an important feature for diagnosis (31, 32). The identification of gene fusions and the use of molecular ancillary testing have improved the classification of RMS by grouping fusion-positive tumors into the alveolar subtype regardless of cytomorphology. However, no differences were observed between fusion-negative ARMS and ERMS (7). In this case, the patient harbors amplifications and losses of multiple genes, involving multiple chromosomes (1, 5, 7, 8, and 19).

Interestingly, the patient also has NTRK1 gene rearrangement. RNA-/DNA-based NGS testing confirms NTRK1 fusion with a partner gene MEF2D. Immunohistochemically, the case is also pan-TRK positive. Therefore, it is necessary to make differential diagnosis between this case and NTRK-rearranged spindle cell tumor. The NTRK-rearranged spindle cell tumor is a rare type of soft tissue tumor with NTRK gene rearrangement as the molecular feature, and is a group of soft tissue tumors defined by molecular genetic features (33). It has a wide spectrum of morphology and tissue classification. IHC often shows co-expression of S-100 and CD34, while lack of other definite differentiations. Its most common features are the phenotype of monomorphic spindle cells, interstitial transparency and infiltrating growth. In this case, IHC of S-100 and CD34 are both negative, moreover, Myogenin, MYO-D1 and Desmin reveal the skeletal muscle differentiation.

Tropomyosin receptor kinases (Trk) are encoded by the neurotrophic tyrosine/tropomyosin receptor kinase (NTRK) genes and belong to the family of tyrosine kinases (34). The Trk family comprises three isoforms, namely TrkA, TrkB, and TrkC, which are encoded by NTRK1, NTRK2, and NTRK3, respectively. In cancer, the most common mechanism of Trk activation involves fusion events that affect NTRK1/2/3. These fusions arise from chromosomal rearrangements between NTRK genes, which include the kinase domains, and various partner genes (35). Currently, there are two drugs approved for treating the NTRK fusion-positive cancers, irrespective of their type: larotrectinib (approved in 2018) and entrectinib (approved in 2019) (36). Involving rearrangements either within or between chromosomes, gene fusions that affect the Trk protein family typically entail the fusion of the 5’ end of a partner gene that contains a dimerization/oligomerization domain with the 3’ region of an NTRK gene that encodes the tyrosine kinase domain. The resulting chimeric gene gives rise to a protein that lacks the TRK ligand binding domain, but retains the tyrosine kinase domain. This fusion protein is associated with oncogenic and transforming potential, which arises from the overexpression and constitutive activation of the TRK kinase domain due to the presence of the dimerization domain derived from the partner gene (37).

In this case, DNA-based NGS results showed MEF2D-NTRK1 (EX5:EX12) gene fusion in tumor cells. Subsequently, we validated NTRK1 gene fusion with RNA-based NGS, which also detected MEF2D-NTRK1 (EX5:EX12) fusion mutation, and the fusion breakpoint sequence was completely consistent with DNA-based NGS. In vertebrates, the myocyte enhancer factor 2 (MEF2) protein family is comprised of four members, MEF2A, B, C, and D, all of which contain a highly conserved MADS-box domain at their N-terminal regions. The MADS-box domain is composed of 55 amino acids and plays a crucial role in recognizing target sequences. The conserved residues within this domain are primarily responsible for binding to DNA sequences rich in A/T and mediating the dimerization of MADS-box proteins. NTRK1 protein is a transmembrane neurotrophic receptor that is found in neural cells and is triggered *via* the binding of its main ligand, nerve growth factor. The NTRK1 comprises an extracellular domain responsible for ligand binding, a transmembrane domain, and an intracellular region harboring the tyrosine kinase domain. Oncogenic activation of NTRK1 leads to autophosphorylation and activation of the MAP-kinase, PI3-kinase and PLC-γ pathways, mediating cell proliferation, survival and differentiation (38). After the rearrangement of MEF2D-NTRK1 (EX5:EX12), the 5’ end of the resulting fusion gene retained the promoter of MEF2D gene to intron 5, and the 3’ end retained the intron 11 of NTRK1 gene to the terminator. The fusion mutation occurs in the intra-codon reading frame, and the fusion protein will retain the tyrosine kinase domain of NTRK1. The protein formed by this fusion mutation retains the MADS-box domain of MEF2D gene at its 5’ end, and the tyrosine kinase domain of NTRK1 at its 3’ end (**Figure 3B**). Moreover, IHC staining of pan-Trk also demonstrated diffusely strong positive in cytoplasm (**Figures 2I, J**). Therefore, the resulting chimera protein is a Trk kinase that is activated in a constitutive manner, irrespective of ligand binding, and has biological functions.

Fusions of the NTRK1 gene are found in lung cancers, colorectal and thyroid cancers, and Glioma, etc (39). It has been reported that 3 cases of NTRK1 fusion were detected in 982 patients with glioma, and one patient had MEF2D-NTRK1 (EX9:EX12) fusion, and IHC detection of pan-Trk showed strong expression (40). Entrectinib (41) and larotrectinib (42) have demonstrated significant efficacy in NTRK fusion-positive tumors. Tadipatri et al. also demonstrated the administration of larotrectinib has resulted in the successful management for remission at 6 months in a high-grade glioneuronal tumor harboring the MEF2D-NTRK1 fusion (43). A patient newly diagnosed low-grade glioneuronal tumor with the BCAN-NTRK1 fusion was treated with entrectinib, 60% tumor reduction at 9 months, then progression at 11 months (44). A 26-year-old male with advanced TPM4-NTRK1 rearranged spindle cell neoplasm and liver, lung and bone metastases, treated with larotrectinib on a continuous 28-day schedule, and showed tumor shrinkage in both visceral and bone lesions after 7 days of treatment (45). ERMS is usually treated primarily by the surgical resection in clinical practice, with adjuvant comprehensive treatment such as radiotherapy, chemotherapy, or targeted drug therapy when necessary to improve patient survival. For this case, the current treatment of this patient is systemic chemotherapy and local radiotherapy after the surgical resection, without the use of NTRK inhibitors. This treatment regimen is currently effective. Targeted drug therapy with NTRK1 fusion will be a very good option if disease progression occurs in the future. NGS assay also identified the NTRK1, EGFR, MET, BRAF and FGFR1 amplifications in this patient. Interestingly, the targeted systemic therapy with larotrectinib was efficacious in a clinical case study, an individual with metastatic esophageal carcinoma was observed NTRK1 amplification (46). The amplification of MET and FGFR, and the activation of bypass signaling molecules including RAS-MAPK/ERK and PI3K-AKT pathways (such as BRAF) are important mechanisms of resistance to tyrosine kinase inhibitors (TKIs) (47). Considering that the patient has a variety of sensitive and drug-resistant mutations involving targeted drugs, whether and how to use targeted drugs in the future need to be further discussed.

Here, we find a rare case of NTRK1 fusion-positive ERMS, which is the first report in literature. In the case, we believe that MEF2D-NTRK1 fusion is a driving mutation and harbors oncogenic and transforming potentials, which is one of the potential pathogeneses. The patient has not been treated with NTRK inhibitors, which is the limitation of the study. Approaches for the identification of cancers driven by NTRK fusions encompass the following tactics: IHC staining of pan-Trk, but NTRK fusion detection by NGS remains the most reliable tool. With NGS application in rare tumors, more NTRK fusion-driven RMS may be found, providing theoretical basis for the follow-up targeted therapy. Therefore, we recommend that all ERMS should undergo the NGS detection with large panel, which can enrich the gene mutation spectrum of ERMS and promote the molecular typing and diagnosis of ERMS. If gene mutation with targeted drug is detected, it will also provide patients with more treatment options.

## Conclusion

Based on the results of morphology, immunology, and genotype analysis, we present a rare ERMS with NTRK1 fusion. With the growing accessibility of NGS analysis, rare tumors are now amenable to management through identifying the targetable molecular markers. Importantly, the oncogenic receptor tyrosine kinase that is abnormally expressed in NTRK-rearranged sarcoma has been proved to have therapeutic targeting, which may improve the prognosis of patients.

## Data availability statement

The original contributions presented in the study are included in the article. Further inquiries can be directed to the corresponding author.

## Ethics statement

The studies involving human participants were reviewed and approved by the Ethics Committee of the Second Xiangya Hospital of Central South University. Written informed consent to participate in this study was provided by the participants’ legal guardian/next of kin. Written informed consent was obtained from the individual(s), and minor(s)’ legal guardian/next of kin, for the publication of any potentially identifiable images or data included in this article.

## Author contributions

X-HL and PZ designed the study. N-ML and S-HJ participated in patient treatment and analyzed clinical data. N-ML and X-HL performed molecular testing and analyzed the data. N-ML and S-HJ drafted the manuscript. X-HL supervised the work and revised the manuscript. All authors contributed to the article and approved the submitted version.
